# Case Report: Multiple mammary carcinomas in a young dog with estrus-associated hyperglycemia: clinicopathological findings and glycemic normalization after ovariohysterectomy

**DOI:** 10.3389/fvets.2026.1930451

**Published:** 2026-09-10

**Authors:** Petru Rosca, Aurelian-Sorin Pasca, Teodor Daniel Hritcu, Stefan Gregore Ciornei, Mihaela Claudia Spataru, Larisa Ivanescu, Luminița Diana Hritcu, Florin Nechifor

**Affiliations:** Faculty of Veterinary Medicine, University of Life Sciences “Ion Ionescu de la Brad”, Iasi, Romania

**Keywords:** canine mammary carcinoma, estrus-associated hyperglycemia, ovariohysterectomy, progesterone-induced insulin resistance, glycemic monitoring, clinicopathological findings, case report

## Abstract

Mammary tumors are among the most common neoplasms affecting female dogs and are typically diagnosed in middle-aged or older animals. The occurrence of malignant mammary tumors in very young dogs is uncommon and may present diagnostic and clinical management challenges. This case report describes a young female dog presenting with multiple mammary nodules accompanied by persistent hyperglycemia detected approximately one to 2 weeks before the onset of estrus. Clinical examination, hematological and biochemical testing, cytological evaluation, surgical excision of the mammary lesions, and histopathological analysis were performed. Histopathology confirmed the presence of multiple mammary carcinomas with different histological patterns. Despite surgical removal of the tumors, blood glucose concentrations remained elevated. Two weeks later, the patient underwent ovariohysterectomy, after which glycemic values gradually decreased and returned to normal within the following weeks. The temporal association between reproductive hormonal status and the observed metabolic alteration suggests a possible hormone-related insulin resistance rather than a direct metabolic effect of the tumors themselves. This case highlights the potential interaction between mammary neoplasia, reproductive hormonal status, and glucose metabolism in dogs and emphasizes the importance of comprehensive clinical and metabolic evaluation in young patients presenting with mammary tumors.

## 1 Introduction

Mammary tumors represent one of the most common neoplasms affecting female dogs and account for a substantial proportion of oncological cases encountered in small animal practice (1–3). Their prevalence varies depending on geographic region, breed, reproductive status, and environmental factors. Epidemiological studies indicate that approximately 50–70% of mammary tumors diagnosed in dogs are benign, while the remaining cases exhibit malignant biological behavior (1–4). Several risk factors have been associated with the development of these tumors, including age, hormonal exposure, breed predis-position, body size, and reproductive history. In particular, prolonged exposure to ovarian hormones such as estrogen and progesterone has been shown to significantly increase the risk of mammary tumor development in intact females compared with spayed dogs (3–6).

Histopathological examination remains the cornerstone for the accurate classification and prognostic assessment of canine mammary tumors. According to the World Health Or-ganization (WHO) classification system, mammary neoplasms may present as simple, complex, mixed, or special histological subtypes, each displaying distinct morphological features and biological behavior (7–9). Several histopathological parameters are used to evaluate tumor aggressiveness and prognosis, including tumor differentiation, mitotic index, cellular pleomorphism, stromal response, and the presence of lympho-vascular invasion (7–10). Well-differentiated tumors with low mitotic activity are generally associated with a more favorable prognosis and reduced metastatic potential compared with poorly differentiated lesions (8–10).

Although mammary tumors are common in female dogs overall, they are typically diagnosed in middle-aged or older animals, most frequently between 8 and 11 years of age (1–3). The occurrence of malignant mammary tumors in very young dogs is considered relatively uncommon and remains poorly documented in the veterinary literature. Early-onset cases may therefore provide valuable clinical insights into tumor development and bio-logical heterogeneity, particularly when multiple histological subtypes are present within the same patient.

In addition to their role in mammary tumor development, reproductive hormones may also influence systemic metabolic regulation. Progesterone, which begins to rise before ovulation as a result of preovulatory luteinization and remains elevated throughout the luteal phase, has been associated with the development of insulin resistance and transient hyperglycemia in susceptible female dogs (11–13). This phenome-non is thought to be mediated by progesterone-induced stimulation of growth hormone production in mammary tissue, which may interfere with insulin signaling and glucose metabolism (11–13). In some cases, these metabolic alterations may persist after the es-trous cycle and gradually resolve following ovariohysterectomy once the hormonal stim-ulus is removed. Despite these recognized endocrine interactions, the relationship be-tween reproductive hormonal status, mammary neoplasia, and glucose metabolism re-mains incompletely documented in veterinary clinical case reports (11–13).

A comprehensive diagnostic approach is essential for the clinical management of mammary tumors. In addition to physical examination and imaging techniques, routine he-matological and biochemical analyses are commonly performed to evaluate the systemic health status of affected patients and to identify possible comorbidities that may influence anesthetic risk and surgical planning (14, 15). However, the metabolic implications of mammary tumors and their interaction with reproductive hormonal status are not routinely explored in clinical reports.

The present case report describes the clinical, cytological, and histopathological findings observed in a young female dog presenting with multiple mammary nodules, together with the temporal evolution of blood glucose concentrations detected prior to estrus and their progression during tumor excision and subsequent ovariohysterectomy. Particular attention was given to the relationship between reproductive hormonal status and glycemic alterations observed in this patient. The report aims to highlight a possible interaction between mammary neoplasia, reproductive hormonal activity, and glucose metabolism in dogs, and to emphasize the importance of integrated clinical, endocrine, and metabolic evaluation in young patients presenting with mammary tumors.

A 2-year-and-4-month-old intact female dog was presented with multiple mammary nodules detected by the owner approximately 2 months before consultation. Clinical examination revealed multiple firm, non-painful mammary masses measuring approximately 6–7 mm in diameter. Initial hematological and biochemical analyses were unremarkable; however, repeated testing performed shortly before estrus revealed persistent hyperglycemia. The patient subsequently underwent surgical excision of the mammary lesions, histopathological evaluation, and ovariohysterectomy, allowing assessment of the temporal relationship between mammary neoplasia, reproductive hormonal status, and glycemic evolution.

## 2 Case presentation

### 2.1 Clinical history and presentation

A 2-year-and-4-month-old intact female mixed-breed dog was presented to the veterinary clinic after the owner noticed several small nodules along the mammary chain approximately 2 months before consultation. According to the owner, the nodules had not shown rapid growth during this period, and the animal did not exhibit signs of pain, discomfort, or behavioral changes.

The dog had no known history of previous systemic disease and had not undergone ovariohysterectomy. At the time of presentation, the owner’s primary concern was the presence of multiple mammary masses detected during routine handling of the animal.

The patient was subsequently admitted for clinical evaluation, laboratory testing, and further diagnostic investigations to determine the nature of the mammary lesions and to establish an appropriate therapeutic plan.

### 2.2 Clinical examination and laboratory findings

At presentation, the patient was in good general condition, with a normal body condition score and physiological parameters within normal limits. Physical examination revealed multiple small nodules located along the caudal mammary glands. The nodules were firm, well-circumscribed, non-painful on palpation, and measured approximately 6–7 mm in diameter. The overlying skin was intact, with no evidence of ulceration, inflammation, or discharge. Regional lymph nodes were palpated and showed no enlargement or signs of pain.

As part of the initial diagnostic work-up, complete hematological and serum biochemical analyses were performed. All hematological parameters were within the physiological reference ranges for the species. Red blood cell count, hematocrit, hemoglobin concentration, platelet count, and leukocyte values did not reveal abnormalities suggestive of systemic inflammatory, infectious, or hematological disorders.

Serum biochemical evaluation, including creatinine, urea, total protein, albumin, globulin, alanine aminotransferase, and alkaline phosphatase, was also within the established reference intervals, indicating normal hepatic and renal function at the time of presentation.

Fasting blood glucose concentration measured during the initial consultation was 84 mg/dL (4.7 mmol/L), which was within the physiological reference range for dogs. No laboratory abnormalities other than the subsequently documented alterations in glucose metabolism were identified during the initial evaluation.

### 2.3 Glycemic monitoring

During the following weeks, while the patient was being prepared for surgical treatment, the owner reported increased water consumption (polydipsia) and more frequent urination (polyuria). Because these clinical signs were suggestive of possible metabolic alterations, biochemical testing was repeated.

At that time, elevated fasting blood glucose concentrations were detected, reaching 280 mg/dL (15.6 mmol/L). According to the owner’s observations, hyperglycemia was first detected approximately one to 2 weeks before the onset of the estrous cycle. These findings prompted continued monitoring of blood glucose concentrations throughout the subsequent clinical management of the patient.

The patient subsequently entered proestrus, characterized by vulvar swelling and vaginal discharge, followed by estrus. Throughout this period, blood glucose concentrations remained elevated despite the absence of other clinical signs suggestive of overt diabetes mellitus.

Blood glucose concentrations were subsequently monitored throughout the diagnostic and perioperative period. After the initial detection of hyperglycemia, fasting blood glucose values progressively increased. During the monitoring period, fasting glycemic concentrations reached 492 mg/dL (27.3 mmol/L) and 520 mg/dL (28.9 mmol/L). These two values were recorded only once and were not reproduced in subsequent measurements. Although the exact cause of these isolated high values could not be definitively established, accidental food intake prior to sampling or transient stress-related hyperglycemia could not be excluded.

In the days preceding surgical excision of the mammary tumors, blood glucose concentrations remained markedly elevated. On the morning of surgery, fasting glycemia measured 401 mg/dL (22.3 mmol/L). During the immediate postoperative period, glycemic values remained high, with measurements of 323 mg/dL (17.9 mmol/L) and 416 mg/dL (23.1 mmol/L) recorded on the first and second postoperative days, respectively.

During the following days, blood glucose concentrations continued to remain elevated, with fasting values ranging between 341 and 387 mg/dL. No favorable glycemic response was observed following surgical excision of the mammary tumors.

Two weeks later, the patient underwent ovariohysterectomy. After this procedure, blood glucose concentrations initially remained elevated for approximately 10 days, with fasting values around 325 mg/dL (18.1 mmol/L). Subsequently, glycemic values began to decrease progressively. Blood glucose concentrations were monitored daily in the morning after fasting for approximately 1 month following ovariohysterectomy.

**Figure 1:**
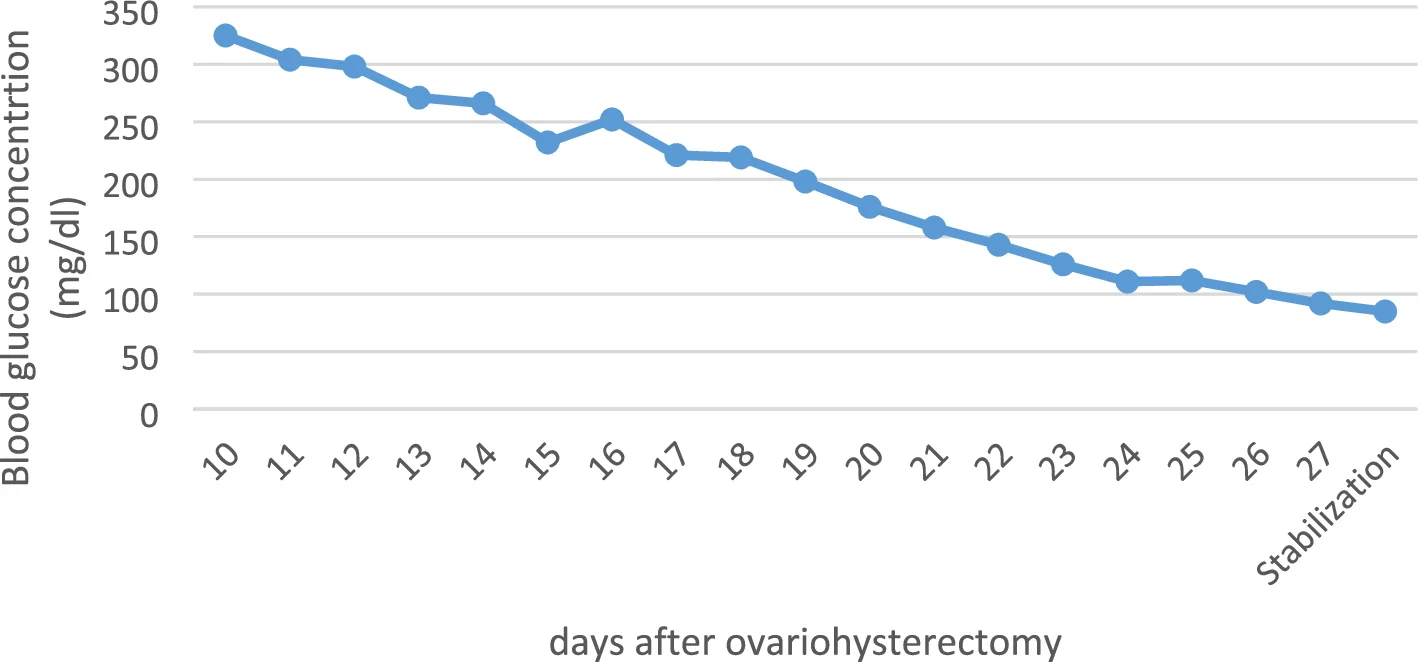
Evolution of fasting blood glucose concentrations following ovariohysterectomy. Blood glucose values were measured daily during the postoperative monitoring period, showing a progressive decline until stabilization within the physiological range.

The daily fasting blood glucose values recorded during the postoperative period are summarized in Table 1, while the progressive postoperative decline in glycemic concentrations is illustrated in Figure 1.

**Table 1:** Daily fasting blood glucose concentrations recorded after ovariohysterectomy.

Day after OHE	Blood glucose (mg/dL)	Blood glucose (mmol/L)
10	325	18.1
11	304	16.9
12	298	16.6
13	271	15.1
14	266	14.8
15	232	12.9
16	252	14.0
17	221	12.3
18	219	12.2
19	198	11.0
20	176	9.8
21	158	8.8
22	143	7.9
23	126	7.0
24	111	6.2
25	112	6.2
26	102	5.7
27	92	5.1
Stabilization	78–91	4.3–5.1

After day 27, fasting blood glucose values stabilized within the physiological.

**Figure 2:**
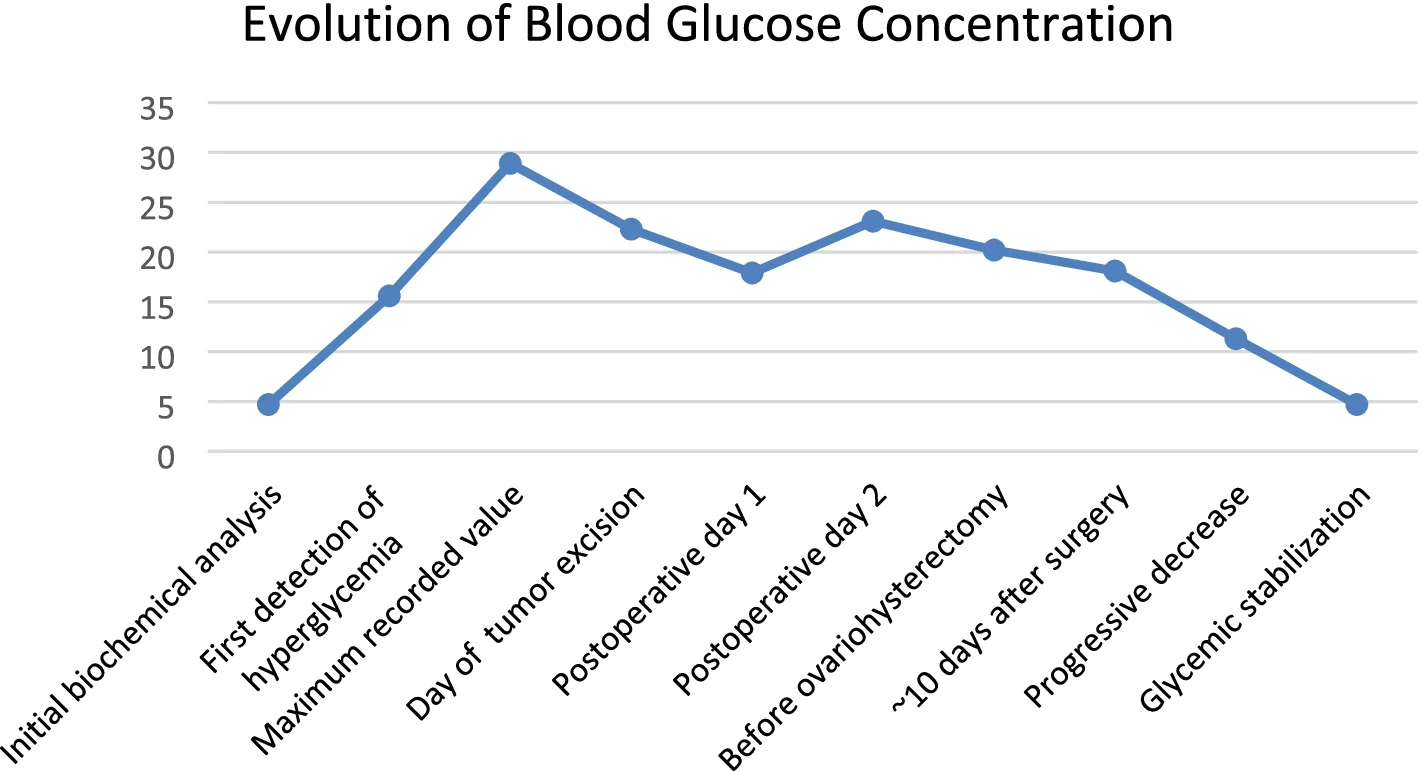
Evolution of fasting blood glucose concentrations during the clinical management of the patient, including the initial biochemical evaluation, detection of hyperglycemia, surgical excision of the mammary tumors, and the period following ovariohysterectomy. A progressive decrease in glycemic values was observed after ovariohysterectomy until stabilization within the physiological range.

A gradual reduction in glycemic values was observed, eventually stabilizing within the physiological reference range, with fasting blood glucose concentrations ranging between 78 and 91 mg/dL (4.3–5.1 mmol/L). The overall evolution of fasting blood glucose concentrations during the clinical management of the patient is summarized in Table 2 and graphically illustrated in Figure 2.

**Table 2:** Evolution of fasting blood glucose concentrations during the clinical management of the patient.

Clinical stage	Time point	Blood glucose (mg/dL)	Blood glucose (mmol/L)	Remarks
Initial consultation	First biochemical analysis	84	4.7	Within reference range
Pre-estrus period	First detection of hyperglycemia	280	15.6	Polydipsia and polyuria reported
Monitoring period	Maximum recorded value	492	27.3	Isolated value
Monitoring period	Maximum recorded value	520	28.9	Isolated value
Preoperative (tumor excision)	Day of surgery	401	22.3	Fasting measurement
Postoperative (tumor excision)	Day 1	323	17.9	Persistent hyperglycemia
Postoperative (tumor excision)	Day 2	416	23.1	Stress-related increase possible
Pre-OHE period	Before ovariohysterectomy	341–387	18.9–21.5	Persistent hyperglycemia
Post-OHE period	~10 days after surgery	~325	~18.1	Hyperglycemia still present
Post-OHE monitoring	Progressive decrease	304 → 102	16.9 → 5.7	Daily monitoring
Follow-up	Stabilization	78–91	4.3–5.1	Within reference range

Blood glucose concentrations were measured in the morning after fasting. Glycemic values were monitored daily for approximately 1 month following ovariohysterectomy.

### 2.4 Diagnostic cytology

Fine-needle aspiration cytology (FNA) was performed prior to surgical excision to obtain a preliminary assessment of the cellular characteristics of the mammary nodules. Cytological samples were collected from two representative lesions using a fine-needle aspiration technique. The smears were air-dried and stained with May–Grünwald–Giemsa stain.

Microscopic examination revealed moderate cellularity composed predominantly of epithelial cells arranged in cohesive clusters and sheets. The cells exhibited relatively uniform morphology, with round to oval nuclei and moderate amounts of cytoplasm. Mild anisocytosis and anisokaryosis were observed, whereas nuclear pleomorphism was limited. Mitotic figures were not detected in the examined smears.

Based on these cytological findings, the lesions were initially interpreted as a benign epithelial mammary proliferation. However, considering the recognized limitations of cytology in differentiating benign from malignant mammary lesions in dogs, definitive diagnosis required histopathological examination of the surgically excised tissues. Subsequent histopathological analysis confirmed the presence of malignant mammary tumors.

**Figure 3:**
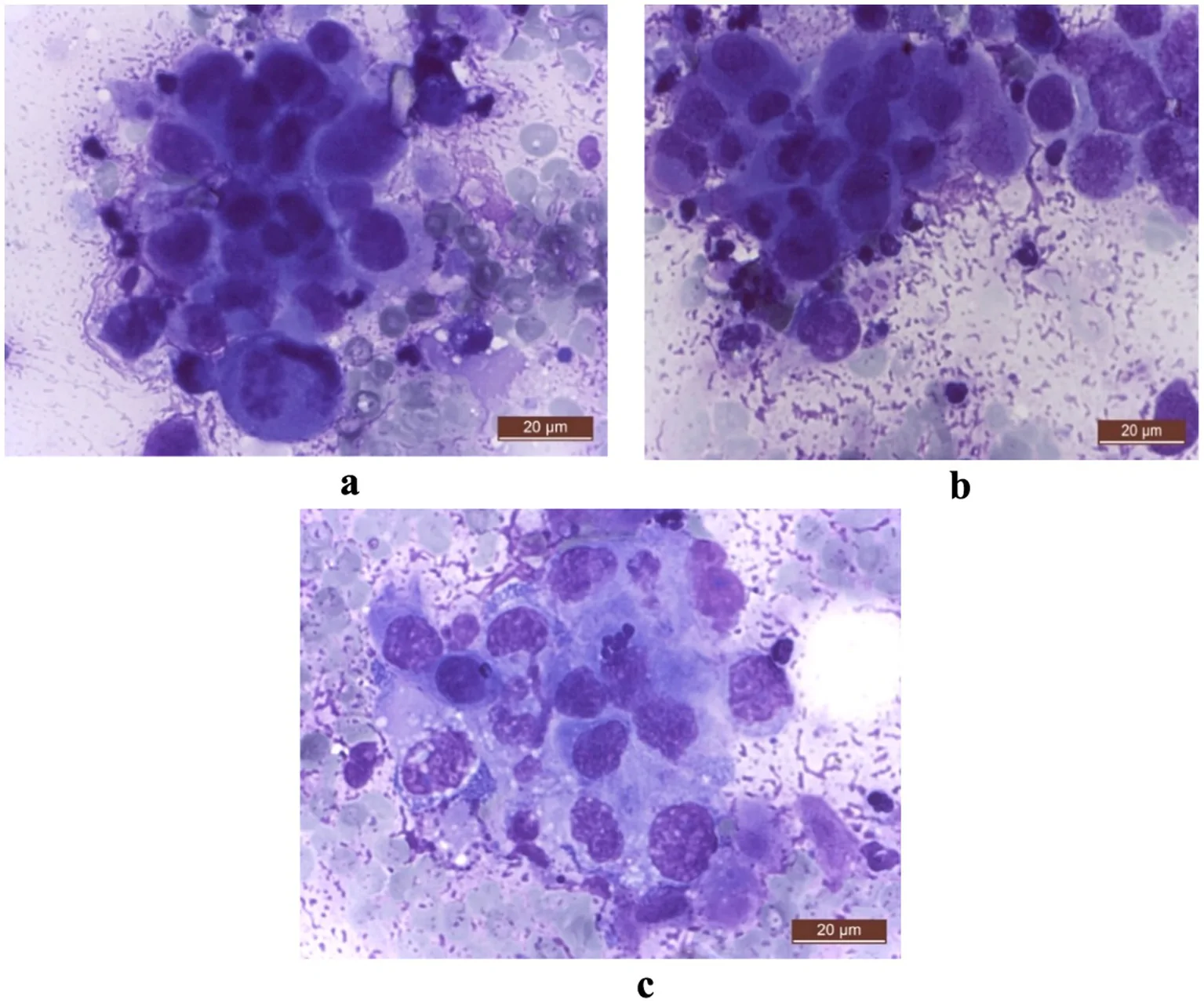
Cytological features of mammary nodules obtained by fine-needle aspiration. **(a–c)** May–Grünwald–Giemsa staining showing cohesive epithelial clusters with minimal cytological atypia and preserved cellular morphology.

Representative cytological findings are shown in Figure 3.

### 2.5 Surgical management

Following clinical evaluation and cytological assessment, surgical treatment was performed under general anesthesia. Because multiple nodules were located within the same mammary segment, a regional partial mastectomy was undertaken to remove all clinically detectable lesions.

A standard skin incision was made over the affected mammary chain, followed by vascular control using bipolar cautery. Blunt dissection was performed to separate the mammary tissue from the underlying abdominal wall and expose the vascular pedicle. The external pudendal artery and vein were isolated and ligated before en bloc excision of the affected mammary segment.

**Figure 4:**
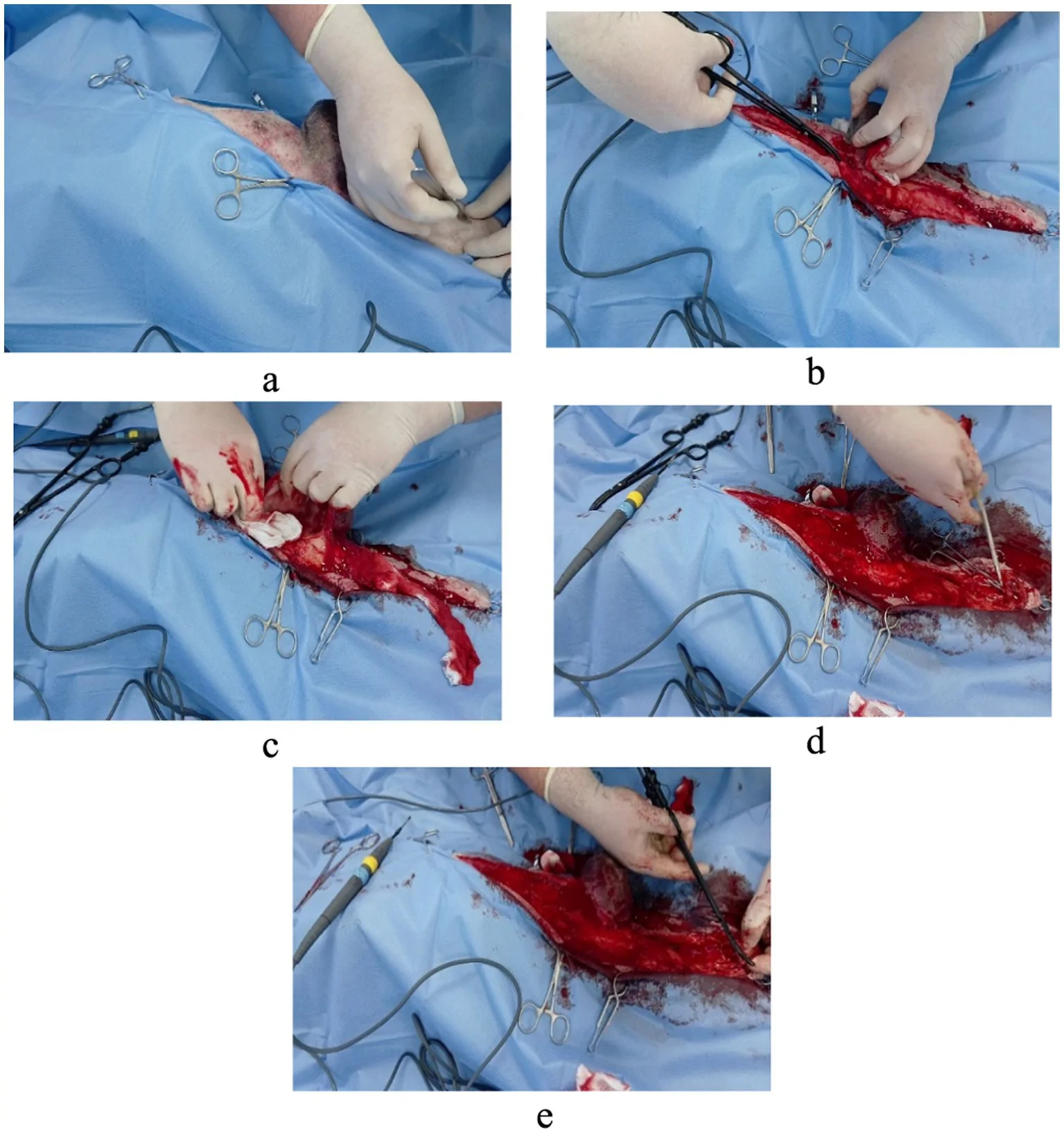
Surgical excision of mammary nodules. **(a)** Skin incision over the affected mammary chain. **(b)** Hemostasis achieved using bipolar cautery. **(c)** Blunt dissection exposing the vascular pedicle. **(d)** Ligation of the external pudendal vessels. **(e)** Surgical field following en bloc excision of the affected mammary segment.

Regional lymph nodes were evaluated intraoperatively and showed no gross abnormalities such as enlargement or altered consistency. All excised tissues, including the mammary lesions and regional lymph nodes, were submitted for histopathological examination. Representative intraoperative photographs illustrating the main surgical steps are shown in Figure 4.

Despite complete surgical excision of the clinically detectable mammary lesions, no improvement in glycemic control was observed during the postoperative period. Considering the persistence of hyperglycemia and the potential influence of reproductive hormonal activity on glucose metabolism, ovariohysterectomy was performed 2 weeks later.

The ovariohysterectomy was completed without intraoperative complications, and the patient recovered uneventfully from anesthesia. Subsequent clinical and glycemic follow-up demonstrated a progressive normalization of fasting blood glucose concentrations, as described in section 2.3.

### 2.6 Gross pathology

Following surgical excision, the mammary tissues were subjected to gross pathological examination. Multiple discrete nodules were identified within the excised mammary segment and were processed individually for histopathological evaluation.

**Figure 5:**
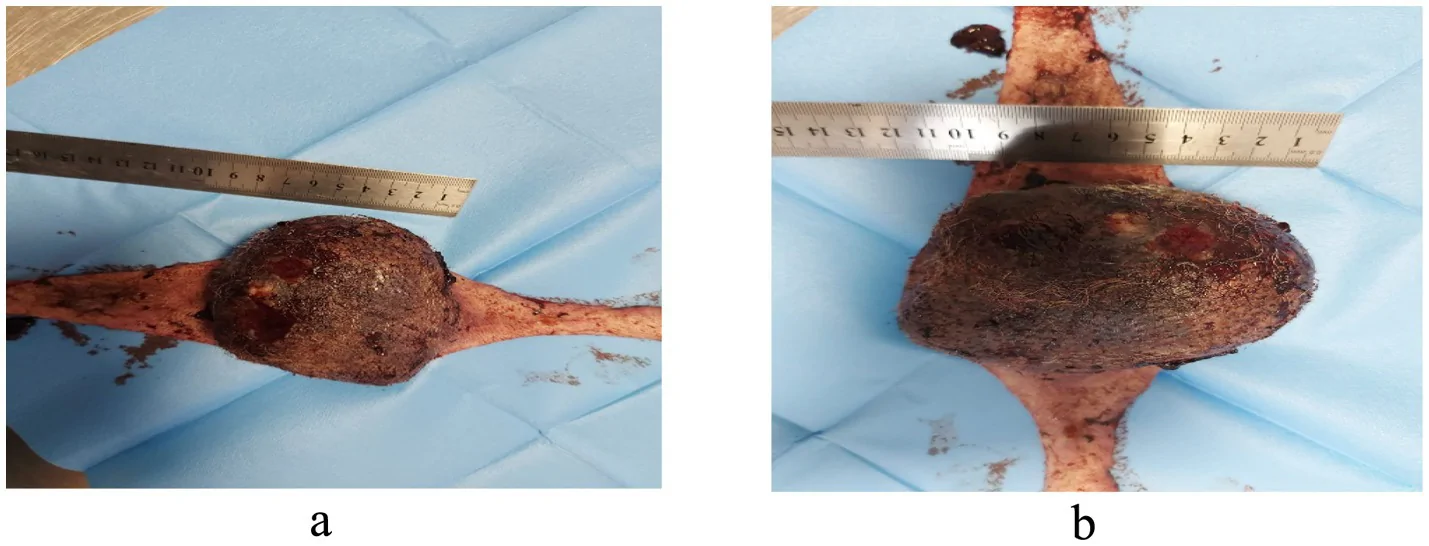
Gross pathological appearance of excised mammary nodules. **(a)** Mammary tissue after surgical excision showing multiple nodular lesions. **(b)** Sectioned mammary nodule demonstrating a well-circumscribed homogeneous whitish-gray cut surface.

The nodules measured approximately 6–7 mm in diameter, were firm in consistency, and appeared relatively well circumscribed from the surrounding mammary tissue (Figure 5a). The external surfaces were smooth, with no evidence of ulceration or infiltration into adjacent structures.

On cut section, the nodules displayed a homogeneous whitish-gray appearance without visible areas of necrosis or hemorrhage (Figure 5b). The lesions appeared clearly demarcated from the surrounding mammary parenchyma.

The adjacent mammary tissue did not show gross evidence of hyperplastic, dysplastic, or inflammatory changes, and no additional nodular lesions were identified. Regional lymph nodes excised during surgery appeared normal on gross examination, without enlargement or alterations in consistency, and were submitted separately for histopathological evaluation.

Representative gross pathological features of the excised mammary nodules are shown in Figure 5.

### 2.7 Histopathological findings

The surgically excised mammary tissues were fixed in 10% neutral buffered formalin, routinely processed, embedded in paraffin, sectioned at approximately 4–5 μm, and stained with hematoxylin and eosin (H&E). Selected sections were additionally stained with Masson’s trichrome and examined by light microscopy.

Histopathological examination revealed the presence of multiple malignant mammary tumors displaying different histological patterns. The neoplastic proliferation consisted predominantly of epithelial cells arranged in tubular and solid structures supported by a variably developed fibrous stroma. The neoplastic cells exhibited moderate cellular and nuclear pleomorphism, with round to oval nuclei, prominent nucleoli, and moderate amounts of eosinophilic cytoplasm.

Occasional mitotic figures were observed, indicating proliferative activity within the tumor tissue. In some areas, the neoplastic cells formed irregular glandular structures consistent with mammary carcinoma. Tumor margins appeared relatively well defined in the examined sections.

Based on the histopathological findings, the lesions were diagnosed as multiple mammary carcinomas with heterogeneous histological patterns.

**Figure 6:**
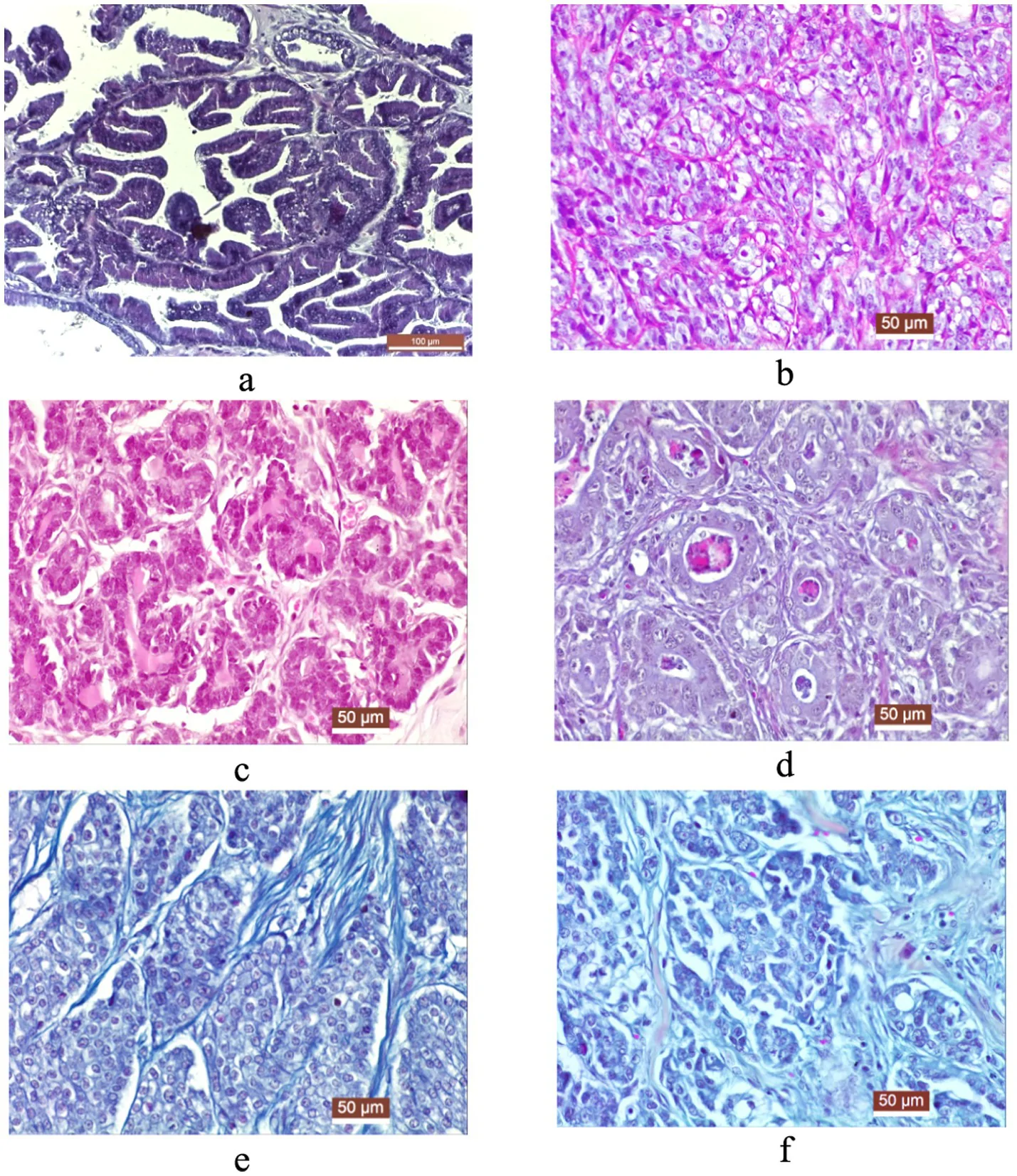
Histopathological features of mammary carcinomas. **(a)** Tubulo-papillary carcinoma with fibrovascular cores (Masson’s trichrome). **(b,c)** Tubular carcinoma composed of irregular tubular structures infiltrating mammary parenchyma (H&E). **(d,e)** Tubular carcinoma with interlobular fibrosis highlighted by Masson’s trichrome staining. **(f)** Complex mammary carcinoma showing epithelial and myoepithelial components (Masson’s trichrome).

Representative histopathological findings are shown in Figure 6.

Histopathological classification was based on routine microscopic examination using hematoxylin–eosin and Masson’s trichrome staining. Additional immunohistochemical or molecular analyses were not performed in this case.

Histopathological evaluation of the regional lymph nodes was not available; therefore, nodal metastatic status could not be determined.

### 2.8 Surgical procedure

Following clinical evaluation and cytological assessment, a regional partial mastectomy was performed under general anesthesia. Because multiple nodules were located within the same mammary segment, complete excision of all clinically detectable lesions within the affected area was undertaken.

The surgical procedure involved a standard skin incision over the affected mammary chain, followed by vascular control using bipolar cautery. Blunt dissection was performed to separate the mammary tissue from the underlying abdominal wall and expose the vascular pedicle. The external pudendal artery and vein were subsequently isolated and ligated before en bloc excision of the affected mammary segment.

Regional lymph nodes were evaluated intraoperatively and did not show gross abnormalities such as enlargement or altered consistency. The surrounding mammary tissue also lacked visible hyperplastic, inflammatory, or ulcerative changes.

All excised tissues were submitted for histopathological examination. Representative intraoperative images illustrating the main surgical steps are presented in Figure 4.

## 3 Discussion

Mammary tumors represent one of the most frequently diagnosed neoplasms in female dogs; however, they are typically reported in middle-aged or older animals, with the highest incidence between 8 and 11 years of age (14, 15). In addition, epidemiological studies and comparative oncology analyses have highlighted the importance of canine mammary tumors as naturally occurring models for human breast cancer and for the study of tumor biology in companion animals (16–18). The occurrence of malignant mammary tumors in very young dogs is considered uncommon and has been only rarely documented in the veterinary literature. In the present case, the patient was only 2 years and 4 months old, which makes the diagnosis of multiple mammary carcinomas particularly unusual. Such early-onset cases may provide valuable insights into the potential role of hormonal influences and other biological factors involved in the pathogenesis of mammary tumors in dogs. To the authors’ knowledge, reports describing multiple mammary carcinomas associated with persistent estrus-related hyperglycemia in a dog as young as the patient described here are extremely limited in the veterinary literature (19, 20). Only isolated reports have described mammary carcinomas occurring in unusually young dogs, whereas progesterone-associated metabolic disturbances have been reported separately. To the best of our knowledge, the combination of multiple mammary carcinomas and persistent estrus-related hyperglycemia observed in the present case appears to be exceptionally rare in the available veterinary literature (21–24).

Reproductive hormones play a central role in the development and progression of canine mammary tumors. Recent advances in comparative oncology have provided a more detailed understanding of the molecular mechanisms linking reproductive hormones with mammary carcinogenesis in dogs. Progesterone exerts its biological effects primarily through progesterone receptor (PR)-mediated signaling, stimulating local production of growth hormone (GH) within the canine mammary gland. In turn, GH activates downstream proliferative pathways and interacts with canonical Wnt signaling and members of the HER receptor family, thereby promoting mammary epithelial proliferation, activation of mammary stem cells, cell survival, and tumor progression. Moreover, GH-mediated impairment of insulin signaling may contribute to peripheral insulin resistance and alterations in glucose homeostasis. Although these molecular pathways were not directly investigated in the present case, they provide a biologically plausible explanation for the temporal association observed between estrus, persistent hyperglycemia, and the normalization of blood glucose concentrations following ovariohysterectomy (20, 21, 23). Estrogen and progesterone have been shown to influence mammary epithelial proliferation and are considered major risk factors in the pathogenesis of mammary neoplasia in intact female dogs (19, 25). Prolonged exposure to ovarian hormones is associated with an increased risk of tumor development, whereas early ovariohysterectomy has been reported to significantly reduce the incidence of mammary tumors. These observations highlight the strong endocrine dependence of mammary tissue and emphasize the importance of hormonal status in the biological behavior of mammary neoplasms. In addition, several studies have demonstrated that the biological behavior of canine mammary carcinomas is influenced by the tumor microenvironment and by molecular and immunohistochemical markers associated with malignant progression, including stromal myofibroblasts, heat shock proteins, and tumor-infiltrating lymphocytes (21–25).

Recent molecular studies have further demonstrated that aberrant activation of the PI3K/AKT/mTOR pathway, dysregulation of TP53, EGFR, HER2, Ki-67, and alterations in E-cadherin expression contribute to tumor proliferation, invasion, metastatic potential, and clinical outcome in canine mammary carcinomas. These biomarkers are increasingly recognized as valuable prognostic indicators and further strengthen the role of canine mammary tumors as translational models for human breast cancer (26–28).

In addition to their role in mammary tumor development, reproductive hormones may also influence systemic metabolic regulation Progesterone, which begins to rise before ovulation as a result of preovulatory luteinization and remains elevated throughout the luteal phase, has been associated with the development of insulin resistance and transient hyperglycemia in susceptible female dogs (12, 20). This phenomenon is thought to be mediated by progesterone-induced stimulation of growth hormone production in mammary tissue, which can interfere with insulin signaling and glucose utilization. At the molecular level, progesterone-induced mammary growth hormone production may interfere with insulin signaling pathways, thereby reducing peripheral insulin sensitivity and impairing glucose utilization. Although the precise molecular events were not investigated in the present case, evidence from experimental and comparative studies suggests that alterations involving the IRS/PI3K/AKT signaling pathway may contribute to the development of transient insulin resistance during periods of increased progesterone exposure. These molecular mechanisms provide a biologically plausible explanation for the reversible hyperglycemia observed in the present case, in which blood glucose concentrations normalized only after ovariohysterectomy removed the hormonal stimulus responsible for the metabolic alterations (11, 12, 23).

As a result, alterations in glucose homeostasis may occur during periods of increased hormonal activity. Such endocrine interactions have been described in association with progesterone-related diabetes mellitus or transient hyperglycemia in intact female dogs (12, 20, 29). Diabetes mellitus in companion animals is widely recognized as a multifactorial disease influenced by endocrine, metabolic, genetic, and environmental factors (30, 31).

In the present case, hyperglycemia was first detected shortly before the onset of the estrous cycle and persisted throughout the diagnostic and surgical management period. Importantly, surgical excision of the mammary tumors did not result in normalization of blood glucose concentrations, suggesting that the neoplastic lesions themselves were unlikely to represent the primary cause of the metabolic disturbance. The sequential surgical interventions provided an opportunity to observe the temporal relationship between tumor removal, persistence of hyperglycemia, and the subsequent normalization of glycemic values following ovariohysterectomy. Blood glucose values remained markedly elevated during the perioperative period and began to decline only after ovariohysterectomy was performed. A progressive reduction in fasting blood glucose values was subsequently observed during the postoperative monitoring period, as illustrated in Table 2 and Figure 2, with glycemic concentrations gradually stabilizing within the physiological range. This temporal association supports the hypothesis that the metabolic alterations observed in this patient were primarily influenced by reproductive hormonal activity rather than by the mammary tumors themselves. This interpretation is also consistent with current comparative oncology concepts indicating that endocrine signaling pathways may influence both tumor biology and systemic metabolic homeostasis through complex interactions between progesterone, growth hormone, insulin signaling, and growth factor networks (21).

These findings highlight the importance of considering endocrine factors when evaluating persistent hyperglycemia in intact female dogs presenting with mammary tumors. Although paraneoplastic metabolic disturbances have occasionally been reported in association with mammary tumors in dogs, such events remain uncommon and may involve tumor-derived growth factors influencing glucose metabolism (32). In the present case, however, the progressive normalization of glycemic values following ovariohysterectomy supports a predominantly hormone-mediated mechanism.

Although the observations presented in this report are limited to a single clinical case, they highlight a potentially relevant interaction between reproductive hormonal status, mammary neoplasia, and glucose metabolism in female dogs. Recent studies have also explored potential associations between metabolic diseases and neoplastic processes in dogs, suggesting that endocrine disorders such as diabetes mellitus may interact with tumor development and progression in complex ways (29, 30, 34, 35). In the absence of endocrine assays or advanced molecular investigations, the exact mechanisms underlying the observed metabolic alterations cannot be fully established. Future investigations integrating endocrine profiling with immunohistochemical characterization and molecular analyses of signaling pathways may provide a more comprehensive understanding of the interactions between reproductive hormones, mammary carcinogenesis, and glucose metabolism in dogs. Such integrative approaches could also strengthen the translational value of spontaneous canine mammary carcinomas as naturally occurring models for hormone-dependent breast cancer in women (21, 24).

Endocrine assays, including serum progesterone, insulin, fructosamine, and growth hormone measurements, were not available in this case and therefore the proposed hormone-mediated mechanism remains inferential rather than definitively demonstrated. Nevertheless, the temporal association between the estrous cycle, persistent hyperglycemia, and the subsequent normalization of glycemic values following ovariohysterectomy suggests a hormonally mediated metabolic disturbance. These findings emphasize the importance of considering endocrine influences when evaluating unexplained hyperglycemia in intact female dogs and support the value of comprehensive clinical and metabolic monitoring in patients presenting with mammary tumors (11). Large veterinary oncology databases have also provided valuable information regarding the distribution of tumors across species, age groups, and anatomical locations in companion animals (33).

However, this report describes a single clinical case, and several limitations should be acknowledged. Endocrine assays, including serum progesterone, insulin, fructosamine, and growth hormone measurements, were not available. In addition, immunohistochemical characterization, histopathological evaluation of the regional lymph nodes, and assessment of surgical margins were not performed or were unavailable, limiting complete oncological staging and molecular characterization of the tumor. Further studies involving a larger number of cases and incorporating endocrine, histopathological, and molecular investigations would be valuable to better clarify the relationship between reproductive hormonal status, mammary neoplasia, and glucose metabolism in dogs.

## 4 Conclusion

This case report describes the occurrence of multiple mammary carcinomas in a 2-year-and-4-month-old intact female dog, an age at which malignant mammary tumors are rarely reported. The case was characterized by persistent hyperglycemia that developed shortly before estrus, remained elevated throughout the diagnostic and surgical management period, and did not improve following excision of the mammary tumors.

A progressive normalization of blood glucose concentrations was observed only after ovariohysterectomy, supporting the hypothesis of a hormonally mediated metabolic disturbance associated with reproductive activity. Although the exact endocrine mechanisms could not be confirmed because hormonal assays were not available, the temporal relationship between estrus, persistent hyperglycemia, and subsequent glycemic normalization following ovariohysterectomy suggests a clinically relevant interaction between reproductive hormonal status and glucose metabolism.

These findings emphasize the importance of considering endocrine influences when evaluating unexplained hyperglycemia in intact female dogs and highlight the value of integrated clinical, pathological, and metabolic assessment in patients presenting with mammary tumors. Further investigations involving larger numbers of cases are needed to better characterize the relationship between reproductive hormones, mammary neoplasia, and glucose homeostasis in dogs.

## Data Availability

The raw data supporting the conclusions of this article will be made available by the authors, without undue reservation.
